# Supplementary material for: Effects of Resistance Training Performed with Different Loads in Untrained and Trained Male Adult Individuals on Maximal Strength and Muscle Hypertrophy: A Systematic Review
Source: Int J Environ Res Public Health. 2021 Oct 26;18(21):11237. doi: 10.3390/ijerph182111237 (PMC8582674; doi:10.3390/ijerph182111237)
Supplement: Supplementary file 1 [file ijerph-18-11237-s001.zip › ijerph-1417416-supplementary.pdf]

SEARCH STRATEGY SYNTAX

EMBASE®
Date: 22 August 2021
Results: 904
Filters applied: article; human
Search Strategy: ('resistance training':ab,ti OR 'strength training':ab,ti OR 'resistance exercise':ab,ti) AND ('high-load':ab,ti OR 'high load':ab,ti OR 'high-intensity':ab,ti OR 'high intensity':ab,ti OR 'heavy loads':ab,ti OR 'low-load':ab,ti OR 'low load':ab,ti OR 'low-intensity':ab,ti OR 'low intensity':ab,ti OR 'volume training':ab,ti OR 'training load':ab,ti) AND ('hypertrophy':ab,ti OR 'muscle size':ab,ti OR 'skeletal muscle enlargement':ab,ti OR 'muscle thickness':ab,ti OR 'muscle mass':ab,ti OR 'muscle fibers, skeletal':ab,ti OR 'muscle, skeletal':ab,ti OR 'growth':ab,ti OR 'cross-sectional area':ab,ti OR 'muscle strength':ab,ti OR 'dynamic strength':ab,ti OR 'dynamic force':ab,ti OR 'maximum repetition':ab,ti OR '1rm':ab,ti OR 'isometric contraction':ab,ti OR 'isometric force':ab,ti OR 'maximal voluntary contraction':ab,ti OR 'mvc':ab,ti OR 'maximal voluntary isometric contraction':ab,ti OR 'mvic':ab,ti)

PUBMED®
Date: 22 August 2021
Results: 1492
Filters applied: humans; journal article; english
Search Strategy: (“resistance training”[Mesh] OR “resistance training”[Title/Abstract] OR “strength training”[Title/Abstract] OR “resistance exercise”[Title/Abstract]) AND (“high-load”[Title/Abstract] OR “high load”[Title/Abstract] OR “high-intensity”[Title/Abstract] OR “high intensity”[Title/Abstract] OR “heavy loads”[Title/Abstract] OR “low-load”[Title/Abstract] OR “low load”[Title/Abstract] OR “low-intensity”[Title/Abstract] OR “low intensity”[Title/Abstract] OR “volume training”[Title/Abstract] OR “training load”[Title/Abstract]) AND (“hypertrophy”[Mesh] OR “hypertrophy”[Title/Abstract] OR “muscle size”[Title/Abstract] OR “skeletal muscle enlargement”[Mesh] OR “skeletal muscle enlargement”[Title/Abstract] OR “muscle thickness”[Title/Abstract] OR “muscle mass”[Title/Abstract] OR “muscle fibers, skeletal”[Mesh] OR “muscle fibers, skeletal”[Title/Abstract] OR “muscle, skeletal”[Mesh] OR “muscle, skeletal”[Title/Abstract] OR “growth”[Mesh] OR “growth”[Title/Abstract] OR “cross-sectional area”[Title/Abstract] OR “muscle strength”[Mesh] OR “muscle strength”[Title/Abstract] OR “dynamic strength”[Title/Abstract] OR “dynamic force”[Title/Abstract] OR “maximum repetition”[Title/Abstract] OR “1RM”[Title/Abstract] OR “isometric contraction”[Mesh] OR “isometric contraction”[Title/Abstract] OR “isometric force”[Title/Abstract] OR “maximal voluntary contraction”[Title/Abstract] OR “MVC”[Title/Abstract] OR “maximal voluntary isometric contraction”[Title/Abstract] OR “MVIC”[Title/Abstract])

WEB OF SCIENCE
Date: 22 August 2021
Results: 1517
Filters applied: articles; english
Search Strategy: (“resistance training” OR “strength training” OR “resistance exercise”) AND (“high-load” OR “high load” OR “high-intensity” OR “high intensity” OR “heavy loads” OR “low-load” OR “low load” OR “low-intensity” OR “low intensity” OR “volume training” OR “training load”) AND (“hypertrophy” OR “muscle size” OR “skeletal muscle enlargement” OR “muscle thickness” OR “muscle mass” OR “muscle fibers, skeletal” OR “muscle, skeletal” OR “growth” OR “cross-sectional area” OR “muscle strength” OR “dynamic strength” OR “dynamic force” OR “maximum repetition” OR “1RM” OR “isometric contraction” OR “isometric force” OR “maximal voluntary contraction” OR “MVC” OR “maximal voluntary isometric contraction” OR “MVIC”) (Topic) and Articles (Document Types) and English (Languages)
